# EMT‐associated bias in the Parsortix® system observed with pancreatic cancer cell lines

**DOI:** 10.1002/1878-0261.70066

**Published:** 2025-06-18

**Authors:** Nele Vandenbussche, Renske Imschoot, Béatrice Lintermans, Lode Denolf, Joachim Taminau, Charlotte Fieuws, Geert Berx, Kris Gevaert, Kathleen B. M. Claes

**Affiliations:** ^1^ Center for Medical Genetics Ghent University Hospital Belgium; ^2^ Department of Biomolecular Medicine Ghent University Belgium; ^3^ Cancer Research Institute Ghent (CRIG) Belgium; ^4^ VIB Center for Medical Biotechnology Ghent Belgium; ^5^ Molecular and Cellular Oncology Lab Inflammation Research Center (IRC) Ghent Belgium; ^6^ Department of Biomedical Molecular Biology Ghent University Belgium

**Keywords:** circulating tumor cells, EMT, liquid biopsy, pancreatic cancer, Parsortix

## Abstract

Pancreatic cancer has a 5‐year survival rate of 12%, highlighting the need for reliable biomarkers for early detection and disease monitoring. Circulating tumor cells (CTCs) have emerged as a promising biomarker, yet their detection remains challenging. This study evaluates the Parsortix® system, a microfluidic device that enriches CTCs based on size and deformability, using pancreatic cancer cell lines. As increasing evidence indicates that during epithelial‐to‐mesenchymal transition (EMT) a cell's deformability increases, we evaluated possible biases by the device. The EMT stage of three pancreatic cancer cell lines, CAPAN‐1, MIA PaCa‐2, and PANC‐1, was assessed to classify them as epithelial, mesenchymal‐like, and hybrid, respectively. Spike‐in experiments showed that epithelial and hybrid phenotypes were more efficiently captured (62.6 ± 18.5% and 65.4 ± 11.1%) than mesenchymal‐like cancer cells (32.8 ± 10.2%). These results were confirmed using an EMT‐inducible breast cancer cell line. Lower recovery rates were found for the cells in a mesenchymal‐like state (31.5 ± 6.4%) than those in an epithelial state (47.56 ± 7.2%). In conclusion, the Parsortix® device may underestimate the number of mesenchymal CTCs.

## Graphical abstract

The Parsortix® system was tested for CTC enrichment using pancreatic cancer cell lines with different EMT phenotypes. Spike‐in experiments showed lower recovery of mesenchymal‐like cells. This was confirmed with an EMT‐inducible breast cancer cell line. These findings suggest that Parsortix® may underestimate mesenchymal CTCs, potentially affecting detection accuracy.

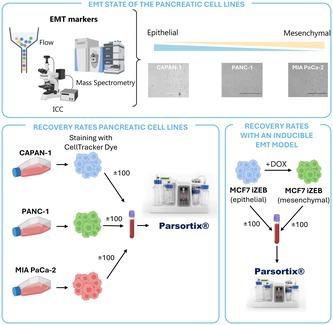

**Abbreviations**
ACN: acetonitrile
APC: allophycocyanin
CA19‐9: carbohydrate antigen 19‐9
CD49f: integrin α6
CDH1: cadherin‐1/E‐cadherin
CDH2: cadherin‐2/ N‐cadherin
CLDN4: claudin 4
CTC: circulating tumor cell
DAPI: 4′,6‐diamidino‐2‐phenylindole
DIA: data‐independent analysis
DMEM: Dulbecco's modified Eagle medium
DOX: doxycycline
E/M: epithelial/mesenchymal
EMT: epithelial‐to‐mesenchymal transition
EPCAM: epithelial cell adhesion molecule
FA: formic acid
FBS: fetal bovine serum
FC: fold change
FDR: false discovery rate
FITC: fluorescein isothiocyanate
FN1: fibronectin‐1
ICC: immunocytochemistry
LC: liquid chromatography
MS: mass spectrometry
NN: neural network
OCLN: occludin
P/S: penicillin/streptomycin
pan‐CK: pan‐cytokeratin
PBS: phosphate‐buffered saline
PBST+: PBS with 0.5% BSA, 0.1% Triton X‐100 and 2% goat serum
qRT‐PCR: quantitative reverse transcriptase polymerase chain reaction
ROCK1: Rho‐associated protein kinase
SD: standard deviation
STR: short tandem repeat
VIM: vimentin
ZEB1: zinc finger E‐box‐binding homeobox 1

## 1 Introduction

Pancreatic cancer is the third‐leading cause of cancer‐related deaths, with a 5‐year survival rate of only 12% [1]. Patients rarely exhibit symptoms at early stages, resulting in more than 50% of patients being diagnosed at an advanced stage [2]. This high incidence of late‐stage diagnoses and subsequent poor prognosis emphasizes the need for reliable biomarkers for early detection, continuous disease monitoring, and clinical decision‐making to improve patient survival [2, 3]. Currently, Carbohydrate Antigen 19‐9 (CA19‐9) is the sole FDA‐approved blood‐based biomarker for pancreatic cancer, despite its low sensitivity and specificity. Other promising biomarkers were suggested, including circulating tumor cells (CTCs), extracellular vesicles, cell‐free RNA, proteins, and cell‐free DNA. CTCs are tumor cells that have detached from the primary tumor and entered the bloodstream, where they can travel to distant organs and potentially form metastases. Their presence in the bloodstream has been associated with poor prognosis in multiple cancer types, for example breast [4], prostate [5], and colorectal cancer [6]. However, detecting and analyzing CTCs remains challenging due to their scarcity [7]. Since its FDA approval in 2004, the CellSearch® system has become one of the most widely used devices for detecting CTCs, relying on immunomagnetic enrichment targeting the epithelial surface marker epithelial cell adhesion molecule (EPCAM) [8]. This device has been extensively tested for multiple cancer types (including breast, prostate, and colorectal cancer); however, for pancreatic cancer, the CTC detection rate is rather low [9, 10]. One possible explanation for this is the epithelial‐to‐mesenchymal transition (EMT) of cancer cells. EMT is a cellular process in which epithelial cells lose their characteristics, such as apical–basal polarity and cell–cell adhesion, and transition into a mesenchymal phenotype, gaining increased invasive and migratory abilities [11, 12]. EMT thus leads to a heterogeneous CTC population, where cells may have an epithelial or mesenchymal phenotype, or exist in an intermediate epithelial/mesenchymal (E/M) state, expressing markers of both states, known as E/M hybrid CTCs. Furthermore, interest in characterizing CTC populations based on epithelial and mesenchymal markers has increased in recent years, as these differentiation states may offer additional information about the tumor stage and patient prognosis. Mesenchymal‐like CTCs were reported to be more invasive with higher metastatic potential than epithelial CTCs [13]. In pancreatic cancer, studies have indicated that the presence of E/M hybrid CTCs and mesenchymal‐like CTCs is correlated with disease progression, whereas the detection of solely epithelial CTCs is not [14, 15].

To capture the entire heterogeneous CTC population, epitope independent size‐based enrichment techniques have become increasingly popular. Among such systems, only the Parsortix® system is FDA cleared, although limited to metastatic breast cancer [16]. It is a semi‐automated, microfluidic device that captures CTCs from whole blood based on their size and deformability. The Parsortix® system therefore seems to be a more appropriate platform for CTC detection as it does not solely rely on an epithelial marker like EPCAM, which is often downregulated during EMT driven cancer progression. However, there is increasing evidence that during EMT the cells' Young modulus of stiffness decreases and deformability increases [17]. Since the Parsortix® system is partly based on deformability, we hypothesized that there might be a bias toward capturing CTCs in the epithelial, less deformable state. The overall aim of our study was to evaluate the performance of the Parsortix® system for the detection of pancreatic cancer cells with different E/M phenotypes.

## 2 Materials and methods

### 2.1 Pancreatic cancer cell lines

Three pancreatic tumor‐derived cell lines were used: CAPAN‐1 (RRID: CVCL_0237), PANC‐1 (RRID: CVCL_0480) and MIA PaCa‐2 (RRID: CVCL_0428). CAPAN‐1 and PANC‐1 cells were received from the Laboratory of Experimental Cancer Research of Prof. Dr. Olivier De Wever (Ghent University Hospital, Belgium). MIA PaCa‐2 cells were purchased from DSMZ GmbH (ACC‐No.: ACC 733) [15]. CAPAN‐1 and PANC‐1 cells were cultivated in Dulbecco's modified Eagle medium (DMEM, 41966029; Thermo Fisher Scientific, Waltham, MA, USA) containing 10% fetal bovine serum (FBS) and 1% penicillin/streptomycin (P/S). MIA PaCa‐2 cells were cultivated in DMEM containing 10% FBS, 1% P/S, and 2.5% horse serum. All cells were incubated at 37 °C and in 5% CO_2_. The cell cultures were regularly checked for mycoplasma contamination with the MycoAlert® Mycoplasma Detection Kit (LT07‐118; Lonza, Basel, Switzerland) according to the manufacturer's guidelines. All cell lines used in this paper were authenticated using short tandem repeat (STR) profiling within the last 3 years [18].

### 2.2 Construction of the MCF7 iZEB1 cellular model

MCF7 cells (RRID: CVCL_0031) were cultured at 37 °C, in 5% CO_2_ in DMEM with 10% FBS, 1% non‐essential amino acids (11140050; Thermo Fisher Scientific), 1% P/S, and 6 ng·mL^−1^ human insulin (I9278‐5ML; Merck Life Science bv, Amsterdam, the Netherlands). The cell culture was checked for mycoplasma contamination with the MycoAlert® Mycoplasma Detection Kit (LT07‐118; Lonza) according to the manufacturer's guidelines. MCF7 cells were transduced with the lentiviral vector pSIN‐hZEB1‐3xHA and selected with puromycin (1 μg·mL^−1^), allowing doxycycline (DOX)‐inducible overexpression of the EMT‐inducing transcription factor ZEB1. The cells were seeded in T25 culture flasks and incubated for 24 h to 20–30% confluence. For each spike‐in experiment, one flask was treated with 1 μg·mL^−1^ DOX hyclate (D5207; Merck Life Science bv) for 72 h; fresh culture medium without DOX was added to the other culture flask. After 72 h, the EMT state of both flasks was determined using microscopy, immunocytochemistry (ICC), and qRT‐PCR to ensure that the cells treated with DOX had switched to the mesenchymal state, while the untreated cells were still in the epithelial state.

### 2.3 Characterization of the cell lines

#### 2.3.1 Immunocytochemistry

CAPAN‐1, PANC‐1, MIA PaCa‐2, and MCF7 iZEB1 cells were used for immunocytochemical analysis. A total of 100 000 cells per well were seeded on chamber slides with eight individual wells (80806; Ibidi, Grafelfing, Germany). MCF7 iZEB1 cells were treated with 1 μg·mL^−1^ DOX hyclate for 72 h. After 3 days, the cells were washed with phosphate‐buffered saline (PBS) and fixed in 4% paraformaldehyde for 10 min. The cells were permeabilized and blocked for 1 h in PBS with 0.5% BSA, 0.1% Triton X‐100, and 2% goat serum (PBST+). The permeabilized cells were washed three times with PBS and incubated overnight at 4 °C with antibodies targeting E‐Cadherin (CDH1) (610182, dilution 1 : 100; BD Biosciences, San Jose, CA, USA), vimentin (VIM) antibodies (ab92547, dilution 1 : 200; Abcam, Cambridge, UK), Zinc finger E‐box‐binding homeobox 1 (ZEB1) antibodies (HPA027524, 1 : 400 dilution; Sigma, Burlington, MA, USA), pan‐cytokeratin (pan‐CK) (ab215838, dilution 1 : 100; Abcam), fibronectin‐1 (FN1) (610078, dilution 1 : 100; BD Biosciences), CD44 (550538, dilution 1 : 100; BD Biosciences), N‐cadherin (CDH2) (33‐3900, dilution 1 : 100; Invitrogen, Carlsbad, CA, USA), claudin 4 (CLDN4) (ab210796, dilution 1 : 100; Abcam) and/or EPCAM (ab213500, dilution 1 : 100; Abcam) diluted in PBST+. The following day, the cells were washed three times with PBS and incubated for 1 h at room temperature with the secondary antibodies: donkey anti‐mouse Alexa Fluor 488 antibody (3553, 1 : 500 dilution; Thermo Fisher Scientific) and/or goat anti‐rabbit Alexa Fluor 568 antibody (A11036, 1 : 2000 dilution; Thermo Fisher Scientific), and 4′,6‐diamidino‐2‐phenylindole (DAPI) (1 : 1000 dilution). All the acquisitions were conducted with a Nikon TiE microscope (Melville, NY, USA) with a spectra X Light Engine (395, 440, 470, 510, 550, and 640 nm). The images were acquired with a DS‐Qi2 Mono Digital Microscope Camera, with ×20/0,75NA CFI Plan Apochromat objective through the interface of the software for nis elements with lasers at 395 nm for DAPI counterstain, 470 nm for CDH1, and 550 nm for VIM. All images were acquired with the same laser power, acquisition time, and gain.

#### 2.3.2 Flow cytometry

The CAPAN‐1, PANC‐1, and MIA PaCa‐2 cells were detached from their T25 culture flasks with EDTA/trypsin and collected in a 15‐mL tube. After centrifugation at 500 ***g*** for 5 min at room temperature, the cell pellets were washed with PBS, transferred to a 1.5‐mL Eppendorf tube, and centrifuged again at 500 ***g*** for 5 min at room temperature. PBS was used to wash the cells between the different steps. The cell pellet was resuspended in 50 μL FACS buffer (PBS with 1% FCS and 1 mm EDTA) with antibodies targeting EPCAM (GTX30708, dilution 1 : 200; GeneTex, Irvine, CA, USA), CDH2 (350812, dilution 1 : 200; BioLegend), CD49f (563706, dilution 1 : 100; BD Biosciences), CDH1 (752477, dilution 1 : 400; BD Biosciences), and CD44 (21270446 s, dilution 1 : 200; Immunotools, Friesoythe, Germany) and incubated for 45 min at room temperature. eBioscience Fixable Viability Dye eFluor 780 (65‐0865‐14, dilution 1 : 1000; Thermo Fisher) was added and incubated for 10 min at room temperature. Cells were centrifuged at 500 ***g*** for 5 min at room temperature, washed, fixed, and permeabilized using the eBioscience Foxp3/Transcription Factor Staining Buffer Set (00‐5523‐00; Thermo Fisher Scientific). Subsequently, cells were centrifuged at 1000 ***g*** for 5 min at room temperature. The cell pellets were resuspended in 50 μL FACS buffer with antibodies targeting VIM (677804, dilution 1 : 400; BioLegend), ZEB1 (3396, dilution 1 : 100; Cell Signaling, Danvers, MA, USA), and CDH1 (755879, dilution 1 : 200; BD Biosciences), and incubated for 45 min at room temperature. Cells were centrifuged at 1000 ***g*** for 5 min and washed. The cell pellets were resuspended in 50 μL FACS buffer with Spark Red 718 goat anti‐mouse IgG antibody (405318, dilution 1 : 400; BioLegend) and BV421 anti‐rabbit (406410, dilution 1 : 100; BioLegend) and incubated for 45 min at room temperature. After centrifugation at 1000 ***g*** for 5 min at room temperature, the cell pellets were resuspended in 200 μL FACS buffer. Flow cytometry was performed with BD FACSymphony A3 (BD Biosciences). Data were analyzed using the flowjo Software (v10.10.0) (Ashland, OR, USA).

#### 2.3.3 qRT‐PCR

Upon treatment of MCF7 iZEB1 cells with 1 μg·mL^−1^ DOX hyclate for 72 h, RNA was purified using the Nucleospin RNA Plus 250 preps kit (740984.250; Macherey‐Nagel, Duren, Germany) according to the manufacturer's instructions. RNA concentrations were measured with the Nanodrop (ND‐2000; Thermo Fisher Scientific). 1500 ng of RNA was reverse transcribed into cDNA using the SensiFast cDNA Synthesis Kit (BIO‐650504; GC Biotech BV, Rijswijk, the Netherlands) according to the manufacturer's instructions. qRT‐PCR was performed with the SensiFast SYBR No‐Rox Kit (CSA‐01190; GC Biotech BV) on a LightCycler 480 system (Applied Biosystems, Waltham, MA, USA). Primers were designed with the primer express software (Perkin Elmer, Springfield, IL, USA) or described in literature. This experiment was done in triplicate. The qBasePLUS (BioGazelle, Zwijnaarde, Belgium) software was applied for data analysis based on the deltaCT method and the geNorm algorithm to define the most stable reference genes. An overview of the qRT‐PCR primers for reference and target genes can be found in Table S1.

#### 2.3.4 Proteome analysis of the pancreatic tumor cells

##### 2.3.4.1 Sample preparation

The cells were seeded in a T75 flask in the recommended culture medium in quadruplicate. When the cell culture reached 70–80% confluence, they were washed with PBS and, while in PBS, the cells were scraped from the bottom of the culture flasks and collected in a 15‐mL tube. The cell suspension was adjusted to 1 × 10^6^ per sample. This suspension was centrifuged at 300 ***g*** for 5 min at room temperature, and the cell pellet was snap frozen and stored at −80 °C until further processing. The samples were prepared in quadruplicate for each cell line. The samples were lysed using the S‐TRAP™ protocol (Protifi, Fairport, NY, USA) and sonicated with a probe sonicator. Lysates were centrifuged at 20 000 ***g*** for 15 min at room temperature and the protein concentration in the supernatants was quantified using the PierceTM BCA Protein Assay (23225 and 23227; Thermo Fisher Scientific), following which proteins were digested on S‐TRAPTM micro spin columns (Protifi). For each sample, 33.7 μg protein was loaded on the columns. After digestion (overnight) and peptide elution, peptide concentrations were measured with DropSense‐16.

##### 2.3.4.2 LC–MS/MS analysis

Peptides were re‐dissolved in 20 μL loading solvent A [0.1% TFA in water : acetonitrile (ACN) (99.5 : 0.5, v : v)] moments before analysis. Two microliters of each sample was injected for liquid chromatography (LC)‐mass spectrometry (MS)/MS analysis on an Ultimate 3000 RSLCnano system in‐line connected to a Q Exactive HF BioPharma mass spectrometer (Thermo Fisher Scientific). Trapping was performed at 20 μL·min^−1^ for 2 min in loading solvent A (0.5% ACN in water 0.1%TFA) on a 20 mm PepMap trapping column (Thermo Fisher Scientific, 300 μm internal diameter, 5 μm beads). The peptides were separated on a 50 cm μPAC Neo™ column (Prototype; Thermo Fisher Scientific), which was kept at a constant temperature of 50 °C. Peptides were eluted by a linear gradient reaching 4.5% MS solvent B (0.1% formic acid (FA) in acetonitrile) after 7 min, 17.5% MS solvent B after 82 min, 35% MS solvent B at 90 min, and 56% MS solvent B after 100 min, followed by a 5‐min wash at 56% MS solvent B and re‐equilibration with MS solvent A (0.1% FA in water). For the first 7 min, the flow rate was set to 500 nL·min^−1^, after which it was kept constant at 300 nL·min^−1^.

The mass spectrometer was operated in data‐independent mode. An isolation scheme was created by the skyline software (Herndon, VA, USA) with 10 *m/z* windows between 400 and 900 *m/z*, with optimized window positioning. MS2 spectra were recorded with a 15 000 resolution, gathering 3 000 000 ions for a maximum of 45 ms. After 30 MS2 spectra, the instrument switched back to MS1 with a resolution of 60 000 after collecting an AGC of 5 000 000 ions with a maximum ion time of 50 ms in a scan range of 375–1500 *m/z*.

##### 2.3.4.3 DIA‐NN MS data analysis

Data analysis was performed with the data‐independent analysis (DIA)‐neural network (NN) (v 1.9) search engine. MIA PaCa‐2 replicate 1 was omitted from the analysis because of its higher variability and lower quality than the other three replicates from that cell line (Table S2 and Fig. S1). Library‐free search was enabled and a new *in silico* library was generated based on the human reference proteome (UP000005640_9606, release version 2024); minimal and maximal fragment *m/z* was set to 200 and 1800, respectively, and contaminants were removed. Peptides and Lib.PG.Qvalue were filtered at a false discovery rate (FDR) of 0.01. *In silico* digestion was set such that it cuts at K* and R* (* denotes K and R can be followed by any amino acid); only peptides with up to two missed cleavages and a length between 7 and 30 amino acids were allowed. Minimal and maximal precursor *m/z* and charge were set to 400 and 1800, 1 and 4, respectively. Cysteine carbamidomethylation was enabled as a fixed modification, oxidation (Methionine), and acetylation (protein N terminus) as variable modifications, with the maximum number of variable modifications set to one. Mass accuracy at the MS2 level was set to 20 and 10 p.p.m. for MS1. Matching between runs was enabled and the neural network classifier was set to single‐pass mode. We continued with the PG.MaxLFQ protein group abundances. The DIA‐NN output was run through an R script to generate a ProteinGroups.txt file. Further data analysis was performed in perseus (v2.0.9.0).

##### 2.3.4.4 Differential analysis and overrepresentation analysis

Differential data analysis was done using the perseus software (v2.0.9.0), the protein intensities were log_2_ transformed and missing values were imputed by minimum values. An ANOVA as well as a pairwise comparison with a two‐sample *t*‐test (S0, FDR = 0.01, 1000 randomizations) between cell lines was performed (Table S3). Significant hits were filtered for a Log_2_ fold change (FC) of at least two. The downregulated proteins and upregulated proteins of the pairwise comparisons were separately subjected to an overrepresentation analysis performed with WebGestalt 2024 (https://www.webgestalt.org/) with as gene pathway database ‘Reactome’. The reference set used was ‘genome protein coding’. Only pathways with false discovery rate‐adjusted *P*‐values < 0.05 were considered (Table S4). The corresponding figures were visualized by graphpad prism (San Diego, CA, USA).

### 2.4 Parsortix® experiments

#### 2.4.1 Cell labelling

A T25 flask of each cell line was prelabeled with one of the following dyes: CellTracker™ Blue CMAC Dye (C2110; Invitrogen), CellTracker™ Green CMFDA Dye (C2925; Invitrogen), or CellTracker™ Deep Red Dye (C34565; Invitrogen). The stock of each dye was dissolved in dimethyl sulfoxide (DMSO) according to the manufacturer's instructions. The cells were stained at a confluence of approximately 70–80%. The cells were first washed with PBS (14190094; Thermo Fisher Scientific) with a pH between 7.0 and 7.3, followed by an incubation period with 4 mL of the PBS/CellTracker™ solution. The working concentration of CellTracker™ Blue was 30 μm, and the incubation time was 30 min (at 37 °C and 5% CO_2_); for CellTracker™ Green, this was 6 μm and 30 min (at 37 °C and 5% CO_2_); for CellTracker™ Deep Red, it was 1 μm and 60 min (at 37 °C and 5% CO_2_). After incubation, the reaction was quenched by adding 5 mL of culture medium.

#### 2.4.2 Spike‐in experiments

The cultured cells were washed with PBS and detached with EDTA/Trypsin. After centrifugation at 194 ***g*** for 10 min, the cells were resuspended in PBS to a concentration of 10 cells·μL^−1^. The LUNA‐II™ Automated Cell Counter (L40002‐LG) was used to count the number of viable cells in suspension and determine the average diameter of the cells. Next, 10 μL (containing on average 100 cells) of each cell type was spiked into the K_2_EDTA tube (367525; BD) containing 10 mL of whole blood from a healthy donor. After spiking in, the blood tubes were slowly inverted at least five times. As a control, 10 μL cell suspension was seeded in a 96‐well black/clear bottom plate (165305; Sanbio BV, Uden, the Netherlands). The 96‐well plate was placed on the bench for at least 1 h, after which the number of cells in each well was counted manually using a Zeiss Axio Observer Z1 Inverted Phase Contrast Fluorescence Microscope (Jena, Germany) using the 10x Air objective and the DAPI, Fluorescein isothiocyanate (FITC) and Allophycocyanin (APC) channels.

Blood separation was done with the Parsortix® device using cassettes with a 6.5 μm gap size and a separation pressure of 99 mbar. The cells trapped in the separation cassette were counted manually using a Zeiss Axio Observer Z1 Phase Contrast Fluorescence Microscope using the 20× Air objective and the DAPI, FITC, and APC channels. The recovery rates were calculated according to the equation below:

$$\text{Recovery rate}\left(\%\right)=\begin{array}{l} \frac{\text{Number of cancer cells in separation cassette}}{\text{Average number of cells counted in the control wells}}\times 100\% \end{array}$$

#### 2.4.3 Statistical analysis

Recovery rates from replicated experiments are reported as mean ± standard deviation (SD). Multiple comparisons between the recovery rates of all cell lines were done with a Friedman test with Dunn correction. Statistical analyses were performed using the graphpad prism (version 10.2.0) and a *P*‐value of < 0.05 was considered significant.

## 3 Results

### 3.1 Characterization of pancreatic cancer cell lines

According to the literature, CAPAN‐1 is an epithelial cell line, while MIA PaCa‐2 has a strict mesenchymal phenotype [19, 20]. PANC‐1 cells were described to display either epithelial‐like or mesenchymal‐like traits depending on the readout assays and culture conditions [19, 20, 21, 22]. Given the conflicting PANC‐1 cell line results, we conducted a thorough characterization of all three cell lines using brightfield microscopy, ICC, flow cytometry, and proteomics to assess their epithelial or mesenchymal phenotype before proceeding with the spike‐in experiments.

#### 3.1.1 Cell morphologies

**Fig. 1:**
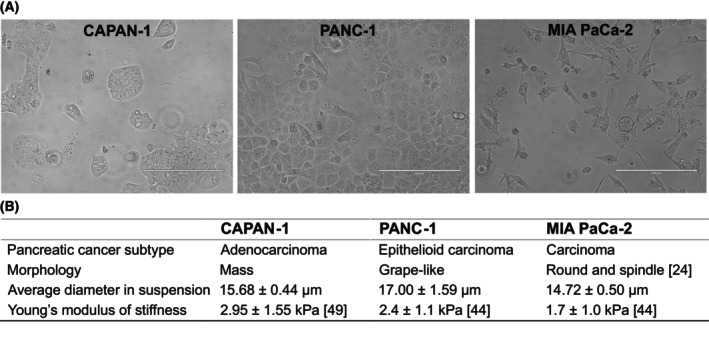
Information about the pancreatic cancer cell lines. (A) Brightfield microscopy of CAPAN‐1 (*n* = 1), PANC‐1 (*n* = 1), and MIA PaCa‐2 (*n* = 1). Scale bar = 200 μm. (B) Table with comparison of pancreatic cancer subtype, morphology, and size (*n* = 6), Young's modulus of stiffness of CAPAN‐1, PANC‐1, and MIA PaCa‐2 [44, 49].

All three cell lines were derived from pancreatic carcinomas; however, they differed in morphology (Fig. 1). EMT is accompanied by morphological changes as epithelial cells are more cobblestone‐like with an apicobasal polarity, while mesenchymal‐like cells have a spindle shape with front‐back polarity. Under 2D adherent culture conditions, CAPAN‐1 cells showed a mass morphology, PANC‐1 cells were grape‐like, and MIA PaCa‐2 cells displayed two different morphologies: round or spindle‐shaped [23, 24]. This indicates that CAPAN‐1 and PANC‐1 cells have an epithelial morphology, while MIA PaCA‐2 cells have a more mesenchymal morphology. Their average cell diameter in suspension was measured before each experiment with the LUNA‐II Cell Counter. On average, CAPAN‐1 cells were 15.68 ± 0.44 μm (range: 5–30 μm), PANC‐1 cells 17.00 ± 1.59 μm (range: 5–31 μm) and MIA PaCa‐2 cells 14.72 ± 0.50 μm (range: 5–25 μm) in size. The size differences between these cell lines were not statistically significant (*P* = 0.0564, Kruskal–Wallis test). For comparison, the Parsortix® separation cassette has a gap size of 6.5 μm and the average CTC diameter varies between 7 and 20 μm [25, 26, 27]. Therefore, the size of the cell lines was not assumed to be a factor that would contribute to a difference in cell retention in the Parsortix® system.

#### 3.1.2 Epithelial/mesenchymal markers

Epithelial cells are characterized by intercellular cell–cell junctions, apical–basal polarity, and interactions with the basement membrane [28]. They express markers like CDH1, EPCAM, and Claudins [29]. During EMT, shifts in gene expression suppress these epithelial traits and markers, and mesenchymal‐like characteristics are promoted. Cells then adopt a fibroblast‐like morphology, undergo structural changes, and gain migratory abilities and invasive properties [28]. In their mesenchymal‐like state, markers like VIM, FN1, CDH2, and CD44 are upregulated [29]. Cells in the hybrid E/M states express both mesenchymal and epithelial markers and traits.

**Fig. 2:**
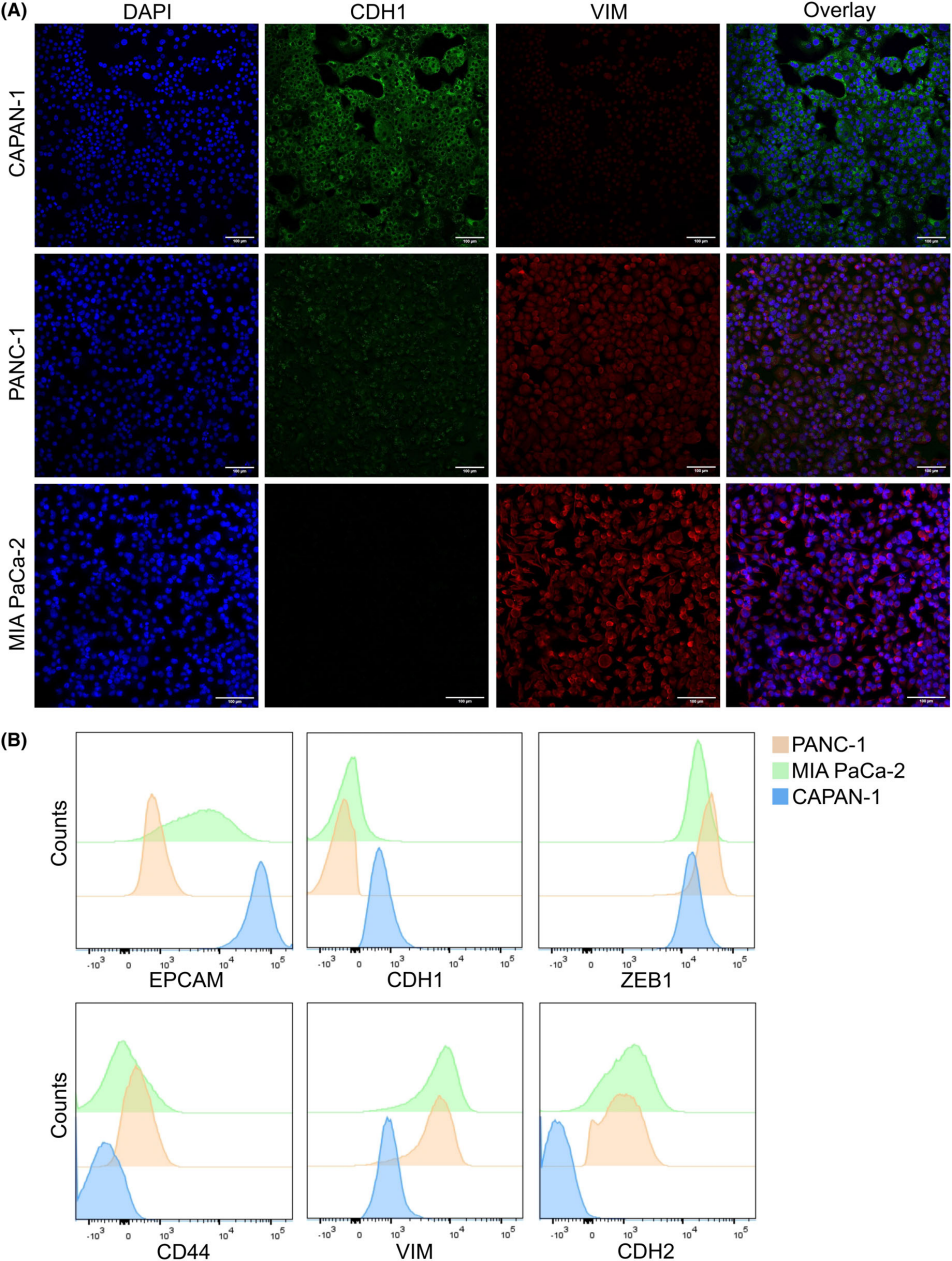
Analysis of the epithelial/mesenchymal phenotype of the pancreatic cancer cell lines. (A) Expression of CDH1 and VIM was evaluated by immunofluorescent staining in the CAPAN‐1 (*n* = 1), PANC‐1 (*n* = 1), and MIA PaCa‐2 (*n* = 1) cells. DAPI was used as a nuclear stain. Scale bar: 100 μm. (B) Expression of EPCAM, CDH1 intracellular, CD44, VIM, CDH2, and ZEB1 was analyzed by flow cytometry.

Initially, we determined the epithelial and/or mesenchymal states of the pancreatic cancer cell lines by assessing the expression of two extensively used markers, CDH1 and VIM, by ICC (Fig. 2A) [30, 31]. In PANC‐1 and CAPAN‐1 cells, CDH1 was primarily detected in the cytoplasm instead of its usual location on the cell membrane, suggesting that the protein is inactive [32]. This was confirmed by flow cytometry, in which intracellular CDH1 and CDH1 on the cell membrane were measured (Fig. 2B and Fig. S2). Intracellularly, CAPAN‐1 cells and, to a lesser extent, PANC‐1 cells were positive for CDH1 expression, while all three cell lines had no or low CDH1 on their cell membrane.

**Fig. 3:**
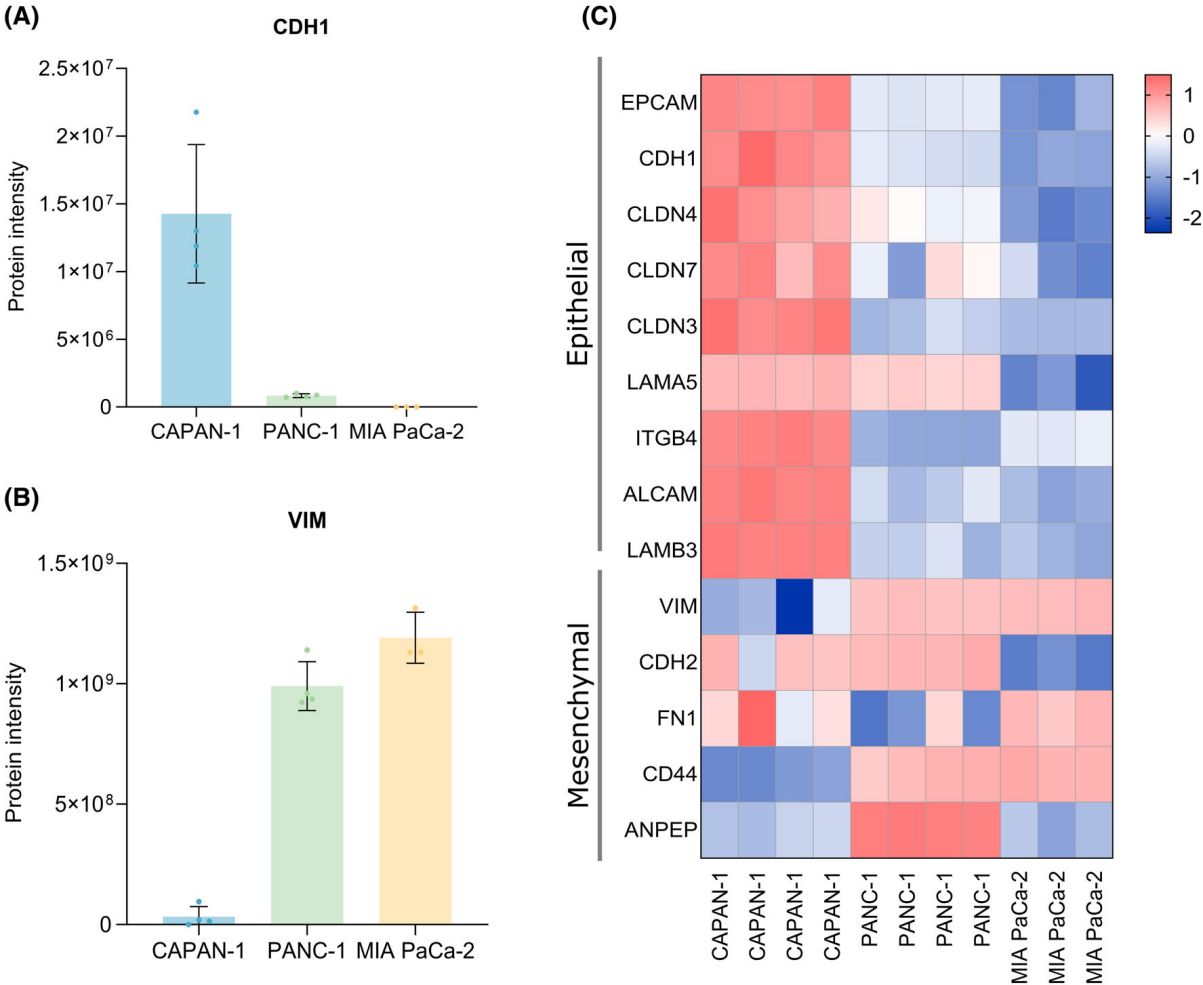
Proteomics data of the CAPAN‐1 (*n* = 4), PANC‐1 (*n* = 4) and MIA PaCa‐2 (*n* = 3) pancreatic cancer cell lines. Protein intensities of (A) the epithelial marker CDH1 and (B) the mesenchymal marker VIM without log_2_ transformation and without imputed values; instead, missing values were replaced with zero. Error bars represent SD. (C) Heatmap of epithelial and mesenchymal markers found in the proteomics data. Values were log_2_ transformed and missing values were imputed from a normal distribution around the detection limit, and the cell values represent normalized intensities through *Z*‐scoring. Red cells indicate a high *Z*‐score, blue cells a low *Z*‐score.

Since inferring an epithelial or mesenchymal phenotype should not happen solely based on VIM and CDH1 [28], additional EMT markers were included in the flow cytometry analysis (Fig. 2B). Proteomics data from the three cell lines was utilized to evaluate a broader panel of E/M markers (Fig. 3). Moreover, additional ICC experiments were done for pan‐CK, EPCAM, CLDN4, FN1, CDH2, and CD44 (Figs S3–S6). A comparison of the results can be found in Table 1. We found some discrepancies between the immunostaining, flow cytometry, and proteomics data. Flow cytometry and proteomics data confirmed the lack of VIM expression in CAPAN‐1 cells, as observed by immunostaining, yet indicated that the levels of VIM in PANC‐1 and MIA PaCa‐2 cells are comparable (Figs 2B and 3B). Furthermore, the mesenchymal CDH2 protein was found in the proteomics data of CAPAN‐1 and PANC‐1 cells (Fig. S5), while in the flow cytometry data, it was found in MIA PaCa‐2 and PANC‐1 cells, which aligns more with what was expected. Flow cytometry detected solely active CDH2 present on the cell membrane. In contrast, ICC staining for CDH2 showed expression in CAPAN‐1 cells but not in PANC‐1 or MIA PaCa‐2 cells. FN1 is a mesenchymal marker but was detected in CAPAN‐1 cells and to a lesser extent in MIA PaCa‐2 cells, and not in PANC‐1 cells with ICC and proteomics (Fig. S3). The mesenchymal marker CD44 was detected in all cell lines with ICC, but with flow cytometry and proteomics only in PANC‐1 and MIA PaCa‐2 cells (Fig. S6). Lastly, the EMT‐associated transcription factor ZEB1 was detected by flow cytometry mainly in MIA PaCa‐2 cells, but not by proteome analysis.

**Table 1:** Overview of the EMT markers in the pancreatic cancer cell lines: CAPAN‐1, PANC‐1, and MIA PaCa‐2. −: not found, no signal; −/+: little to no signal; +: present in cells; ++: high expression; /: not included in experimental set up. Flow, flow cytometry; ICC, immunocytochemistry; MS, proteomics.

	CAPAN‐1			PANC‐1			MIA PaCa‐2		
	ICC	Flow	MS	ICC	Flow	MS	ICC	Flow	MS
Epithelial									
CDH1	++	+	++	+	−/+	+	−	−	+
EPCAM	++	++	++	−	+	−/+	−	−	−
CLDN4	++	/	+	−/+	/	++	−/+	/	−
pan‐CK	++	/	++	−	/	+	−	/	−/+
Mesenchymal									
VIM	−	−	−	+	++	++	++	++	++
CD44	+	−	−/+	+	−/+	++	++	+	++
CDH2	−/+	−	+	−	++	++	−	++	−
FN1	+	/	+	−	/	−/+	−/+	/	+
ZEB1	/	−	−	/	−/+	−	/	+	−

Based on the molecular markers along with their morphology, we conclude that CAPAN‐1 cells exhibit an epithelial phenotype, MIA PaCa‐2 cells are mesenchymal‐like, and PANC‐1 cells are in transition between the two states, displaying an E/M hybrid phenotype. Therefore, these three pancreatic cell lines seem to represent the different CTC subtypes found in pancreatic cancer patients.

### 3.2 Characterization of an inducible EMT model: the MCF7 iZEB1 cell line

**Fig. 4:**
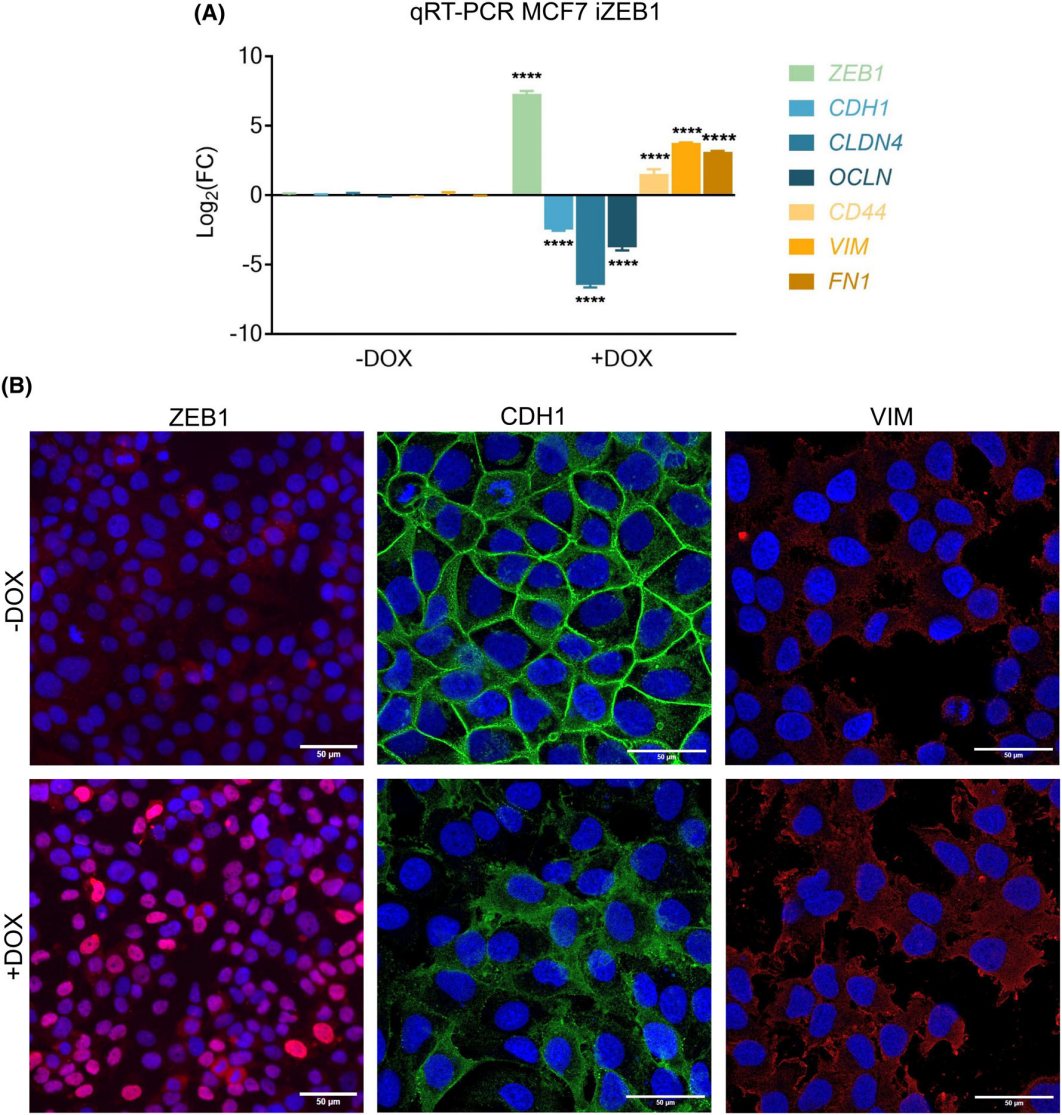
Analysis of the epithelial/mesenchymal phenotype of MCF7 iZEB1 cells after 72 h of DOX induction. (A) Log_2_(FC) values of mRNA expression levels of ZEB1, epithelial (CDH1, CLDN4, OCLN), and mesenchymal (CD44, VIM, FN1) markers in doxycycline (DOX)‐induced (*n* = 3) versus noninduced MCF7 iZEB1 cells (*n* = 3). A multiple comparison between markers in the −DOX and +DOX groups with an ANOVA test with Šídák's correction was performed (****P < 0.001). Error bars represent SD. (B) Expression of ZEB1, CDH1, and VIM was evaluated by immunofluorescent staining in the MCF7 iZEB cells (*n* = 3). DAPI (blue) was used as a nuclear stain. Scale bar: 50 μm.

The MCF7 iZEB1 cells were treated with DOX for 72 h, followed by qRT‐PCR to analyze the expression of *ZEB1* and several EMT markers. DOX treatment induced *ZEB1* expression at the mRNA level, reduced the RNA levels of epithelial markers *CDH1*, *CLDN4*, and Occludin (*OCLN*), and upregulated mesenchymal markers *CD44*, *VIM*, and *FN1* (see Fig. 4A). Immunocytochemistry was then performed on DOX‐treated cells to confirm these findings at the protein level. ZEB1 expression was observed in the DOX‐treated cells, with no detectable leaky expression in noninduced cells. This was accompanied by the loss of CDH1 at the plasma membrane and increased vimentin levels (see Fig. 4B), which proves that the cells underwent EMT after DOX treatment.

### 3.3 Parsortix® experiments

**Fig. 5:**
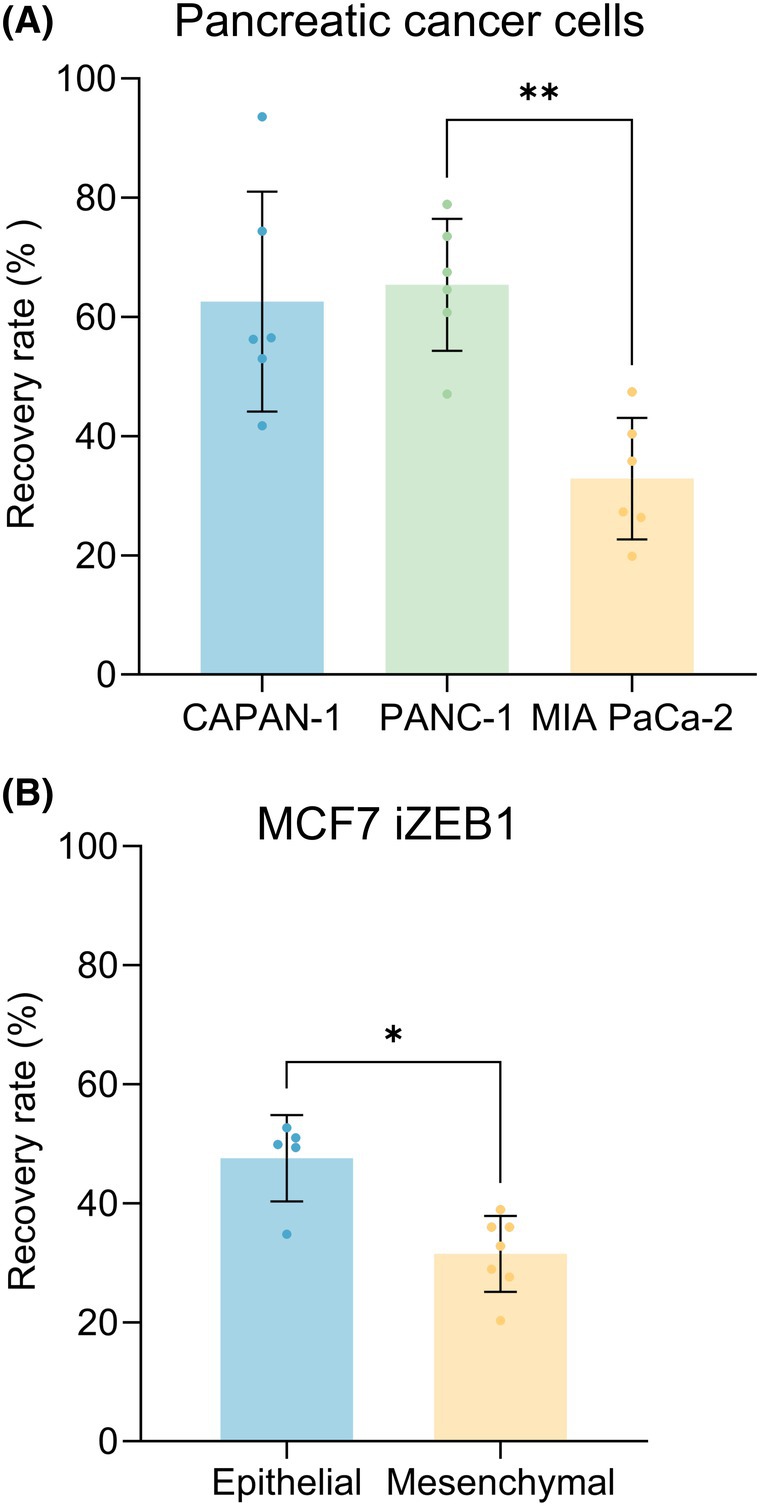
Parsortix® system recovery rates (A) for the pancreatic cancer cell lines: CAPAN‐1: 62.6 ± 18.5% (*n* = 6), PANC‐1: 65.4 ± 11.1% (*n* = 6), and MIA PaCa‐2: 32.8 ± 10.2% (*n* = 6). A multiple comparison between all cell lines with a Friedman test with Dunn correction was performed (**P < 0.01) (B) for the MCF7 iZEB1 cells treated with doxycycline (mesenchymal phenotype) and without doxycycline (epithelial phenotype); Epithelial (*n* = 5): 47.56 ± 7.2% and mesenchymal (*n* = 7): 31.5 ± 6.4%. Mann–Whitney test between groups (*P < 0.05). Error bars represent SD.

The capability of the Parsortix® system to enrich pancreatic epithelial, mesenchymal, and hybrid cancer cells was evaluated through spike‐in experiments using the three pancreatic cancer cell lines. Approximately 100 cells from each cell line were spiked into 10 mL of healthy donor blood, and individual cell line recovery rates were calculated (see Fig. 5A). The counts of the control wells and calculations of the recovery rates can be found in Table S5. PANC‐1 and CAPAN‐1 cells were found to have about a two‐fold higher recovery ratio than MIA PaCa‐2 cells (mean recovery rate ± SD; CAPAN‐1: 62.6 ± 18.5%, PANC‐1: 65.4 ± 11.1%, and MIA PaCa‐2: 32.8 ± 10.2%). These results indicate that the mesenchymal pancreatic tumor cells (MIA PaCa‐2) are less effectively enriched by the Parsortix® system compared with epithelial or hybrid cells (CAPAN‐1 and PANC‐1).

To rule out that the difference in recovery rate in the previous experiment could be due to inherent deformability differences between the pancreatic cell lines, the Parsortix® experiment was repeated using the MCF7 iZEB1 EMT cellular model. The spike‐in experiment was done using the MCF7 iZEB1 cells in the epithelial and the mesenchymal‐like (DOX‐induced) state. Again, we found that cells with a mesenchymal‐like phenotype had a significantly lower recovery rate (see Fig. 5B and Table S5): MCF7 iZEB1 cells in the epithelial state had a recovery rate of 47.56 ± 7.2%, while MCF7 iZEB1 cells in the mesenchymal‐like state had a recovery rate of 31.5 ± 6.4%. The MCF7 iZEB1 cells did not differ significantly in size; the epithelial cells were, on average, in suspension 16.81 ± 1.1 μm and the mesenchymal‐like cells were 16.14 ± 0.6 μm. So, the only difference between the two groups here is the EMT state. Therefore, this experiment confirms that fewer mesenchymal‐like cancer cells are retained during enrichment with the Parsortix® system compared with epithelial cancer cells.

## 4 Discussion

### 4.1 Characterization of pancreatic cancer cell lines

We started by thoroughly characterizing all three pancreatic cell lines to assess their epithelial or mesenchymal phenotype. There were some discrepancies between the immunostaining, flow cytometry, and proteomics data. Differences in protein localization, posttranslational modifications, and detection sensitivity across techniques can contribute to these variations. Proteomics measures total protein levels, including intracellular and inactive forms, which may not always correlate with the expression of functional proteins. On the contrary, immunocytochemistry and flow cytometry depend on antibody specificity and staining conditions, which can sometimes lead to inconsistent results. The antibody used in the ICC staining for CDH2 lacked specificity (Fig. S3), making these results less reliable. Additionally, EMT is a dynamic and context‐dependent process, with cells existing along a spectrum rather than in clearly distinct epithelial or mesenchymal states. The heterogeneity within cell populations, the presence of hybrid E/M phenotypes, and variations in experimental conditions further complicate phenotype classification. This highlights the complexity of defining the EMT state of cancer cells and underscores the challenges associated with the use of a single detection method to classify epithelial or mesenchymal phenotypes.

Note that our categorization of the PANC‐1 cell line contradicts some other studies, which were however often only based on CDH1 and VIM expression evaluated by western blots or CDH1 expression on the cell membrane [19, 20]. Ungefroren et al. [22] suggested that cultures of PANC‐1 cells could simultaneously contain epithelial and mesenchymal‐like cells, and cells in transition. Our immunostainings indeed revealed that some PANC‐1 cells only expressed CDH1 and others only VIM; however, the majority of PANC‐1 cells were positive for both markers (Fig. 1A).

### 4.2 Parsortix® experiments and deformability

Our spike‐in experiments demonstrate that the Parsortix® system enriches epithelial and E/M hybrid cancer cells more efficiently than mesenchymal‐like cancer cells. This trend was observed in both the pancreatic cancer cell lines and the EMT‐inducible model. Hvichia et al. [33] observed a similar trend in their spike‐in experiments with the Parsortix® system. They evaluated five cell lines from different cancer types: PANC‐1, A375, PC3, A549, and T24 cells. All had a similar recovery rate between 60% and 70%, except for the T24 cells, which had an average recovery rate of 42%. The authors initially attributed this difference to cell size; however, all cells were on average larger than the 10 μm gap of the Parsortix® separation cassette. Yet, the difference in cell recovery rate could also be linked to their E/M phenotype since this is probably associated with their deformability [17]. Literature describes the cell lines with higher recovery rates as having an epithelial or E/M hybrid phenotype, whereas T24 cells are characterized by a mesenchymal‐like phenotype [34, 35, 36, 37]. This is in agreement with our findings and indicates that the Parsortix® system, like the CellSearch® system, seems biased toward epithelial and E/M hybrid cancer cells.

Overall, epithelial cancer cells have a highly structured, rigid cytoskeletal network that is often incompatible with cell deformability, while mesenchymal cancer cells acquire dynamic cytoskeleton properties and are more deformable, which supports successful metastasis [38, 39, 40, 41]. Bagnall et al. [39] proved that mesenchymal cancer cells were more deformable and passed faster through a microchannel compared with epithelial tumor cells. Since the Parsortix® system enriches CTCs based on size and deformability, it makes sense that the mesenchymal, often the more deformable cells are less efficiently retained [33]. Since MIA PaCa‐2 cells had a lower recovery rate than CAPAN‐1 and PANC‐1 cells, we suspected pathways related to deformability to be downregulated in these cells. Therefore, differentially expressed proteins from the MS data were functionally annotated via a REACTOME pathway overrepresentation analysis (Fig. S7). An overlap was found in pathways that were significantly underrepresented in MIA PaCa‐2 cells compared with PANC‐1 and CAPAN‐1 cells, like the ‘Rho GTPase cycle’. This pathway regulates the actin cytoskeleton and influences cell migration, cell adhesion, cell division, and establishes cellular polarity. The RhoA pathway is the most well‐known pathway in this group, responsible for contractility of cells [42]. RhoA activates Rho‐associated protein kinase (ROCK), which leads to activation of downstream proteins like myosin II, ultimately leading to an increase in cell contractility, cytoskeletal rigidity and Young's modulus of stiffness, and thus a decrease in cell deformability. This increased contractility in PANC‐1 cells compared with MIA PaCa‐2 cells could explain the difference in recovery rates with the Parsortix® system.

In breast cancer, a proven correlation exists between the deformability of CTCs and the severity of the cancer [43]. The higher the grade and stage of the cancer, the higher the CTC deformability. However, there has also been evidence indicating the opposite, stating that stiffer breast cancer cells are more invasive. Nguyen et al. [44] suggested that the relationship between the stiffness of cancer cells and their invasive potential may depend on the type of cancer. While many studies identify more deformable breast and ovarian cancer cells as more invasive, stiffer lung cancer and pancreatic cancer cells have greater invasive potential. For example, there has been evidence indicating that PANC‐1 cells, while having a higher Young's modulus, are slightly more invasive than MIA PaCa‐2 cells [44]. The higher Young's modulus in PANC‐1 cells explains the higher recovery rate with the Parsortix® system. However, whether the cells are more invasive or not, it should be kept in mind that when using the Parsortix® system, a part of the mesenchymal, deformable CTCs population is lost during enrichment.

Nitschke et al. [45] analyzed 19 blood samples from palliative pancreatic cancer patients with the Parsortix® system and detected CTCs in only 26.3% of the patients, with on average 3.6 CTCs per 7.5 mL of blood. In contrast, Zhu et al. [46] analyzed 40 patients with stage I to IV pancreatic cancer by density gradient and CD45 depletion and found CTCs in 75% of the patients, with on average 33 CTCs per 7 mL of blood. So, it seems that other enrichment methods, like immune cell depletion, result in higher recovery rates, perhaps making them the better choice for studying pancreatic cancer CTCs.

### 4.3 Limitations of the study

One needs to keep in mind that an epithelial or mesenchymal cellular phenotype is not ‘locked’ and cells, even in culture, can switch from one state to another or be in transition between these states [28]. We determined the phenotype of cells grown under 2D culture conditions and it is thus possible that in a different environment, cells can switch to another phenotype. In this respect, ideally, the epithelial/mesenchymal state should also be assessed through a combination of cellular properties, like their invasive and migrative capacities besides multiple molecular markers [28]. However, since the blood samples were immediately processed after spike‐in, we expected that the cells had limited time to undergo significant changes in their EMT state.

Variability in recovery rates between experiments was observed, which can partly be explained by the inexact spike‐in procedure, manual counting of cells in the separation cassette, and variations in cell size within the culture. However, we suspect a significant portion of this variability may be inherent to the Parsortix® device itself. For longitudinal counting of CTCs in blood samples during patient treatment, a robust enrichment system is crucial. To accurately assess the variability introduced by the Parsortix® device and other CTC enrichment techniques, the spike‐in process should be optimized to minimize discrepancies between the actual and expected number of spiked cells. Standardizing this step would allow for a more precise evaluation of the device's consistency and performance.

The use of prestained cells for detection does not fully account for the real heterogeneity of CTCs observed in patients. Different cell lines were added to a single blood sample to mimic the subtypes of CTCs in patient samples. Despite this, the lack of CTC clusters and interactions with immune cells makes our samples less comparable to patient samples. Consequently, the performance of the Parsortix® system might differ between the simulated spiked‐in samples and real clinical samples. However, we assume that in reality, CTC recovery rates will be even lower, as the cancer cells used here are on average larger in size and prestained, thus easier to track down in the separation cassette.

With the Parsortix® system, it is also possible to use fixed blood samples (e.g., Transfix® or CellSave® tubes), while we focused on EDTA blood tubes. This way the blood cells and consequently the CTCs are fixed and less deformable, thus increasing the recovery rate [47]. A drawback of this method is the large restriction for downstream analysis as the cells are not viable anymore, which excludes CTC expansion in cell cultures and makes RNA sequencing and mass spectrometry more challenging. However, if the sole focus is CTC counting, it may be recommended to use fixed blood samples, yet CTCs could provide more information on the disease stage and tumor heterogeneity if analyzed more extensively after enrichment.

## 5 Conclusions

In this study, the E/M phenotypes of three pancreatic cancer cell lines, CAPAN‐1, PANC‐1, and MIA PaCa‐2, were extensively characterized with ICC, flow cytometry, and proteomics. The CAPAN‐1 cells were found to be epithelial, MIA PaCa‐2 cells mesenchymal‐like, and PANC‐1 cells E/M hybrid. The main goal was to assess the effectiveness of the Parsortix® system in enriching these cancer cells, utilizing whole blood samples spiked with the pancreatic cancer cell lines representing a heterogeneous CTC population. The spike‐in results indicated that while epithelial and E/M hybrid phenotypes are efficiently captured (62.6 ± 18.5% and 65.4 ± 11.1%) with the Parsortix® system, mesenchymal‐like cancer cells exhibit lower recovery rates (32.8 ± 10.2%), likely due to their increased deformability. These findings were confirmed with an EMT‐inducible breast cancer cell line: a significantly lower recovery rate was found for MCF7 cells in the mesenchymal‐like state than those in the epithelial state. This shows that the E/M state of cells influences their enrichment efficiency by the Parsortix® system.

Thus, although the Parsortix® system addresses some limitations of other methods, our findings indicate that it tends to underestimate the total number of circulating cancer cells and is more efficient at enriching epithelial and E/M hybrid phenotypes than mesenchymal‐like cells. Future research should focus on refining the technology to minimize CTC loss and to fully capture the heterogeneity of CTC populations, which is essential for translating these findings into clinical practice.

## Conflict of interest

The authors declare no conflict of interest.

## Author contributions

NV contributed to the conceptualization, methodology, formal analysis, project administration, investigation, visualization, and writing—original draft and editing. RI contributed to the investigation, formal analysis, methodology, visualization, and writing—review and editing. BL contributed to the investigation. LD contributed to the investigation, data curation, and validation. JT contributed to the investigation and methodology. CF contributed to the conceptualization, and writing—review and editing. GB contributed to the resources, and writing—review and editing. KG and KBMC contributed to the conceptualization, supervision, funding acquisition, project administration, and writing and editing. All authors gave final approval of the completed version.

## Peer review

The peer review history for this article is available at https://www.webofscience.com/api/gateway/wos/peer‐review/10.1002/1878‐0261.70066.

## Supporting information


**Fig. S1.** Quality control of the label‐free quantification mass spectrometry data of all the pancreatic cancer cell lines CAPAN‐1, PANC‐1 and MIA PaCa‐2 with the first replicate of MIA PaCa‐2 omitted.
**Fig. S2.** CDH1 expression of extracellular CDH1 on the CAPAN‐1, PANC‐1 and MIA PaCa‐2 pancreatic cancer cell lines by flow cytometry.
**Fig. S3.** Expression of pan‐cytokeratins (pan‐CKs) in the CAPAN‐1, PANC‐1 and MIA PaCa‐2 pancreatic cancer cell lines.
**Fig. S4.** Analysis of expression of mesenchymal/epithelial markers fibronectin‐1 (FN1) and EPCAM in the CAPAN‐1, PANC‐1 and MIA PaCa‐2 pancreatic cancer cell lines.
**Fig. S5.** Analysis of expression of epithelial/mesenchymal markers claudin‐4 (CLDN4) and N‐cadherin (CDH2) in the CAPAN‐1, PANC‐1 and MIA PaCa‐2 pancreatic cancer cell lines.
**Fig. S6.** Analysis of expression of mesenchymal marker CD44 in the CAPAN‐1, PANC‐1 and MIA PaCa‐2 pancreatic cancer cell lines.
**Fig. S7.** Differential expression analysis between the pancreatic cell lines: CAPAN‐1, PANC‐1 and MIA PaCa‐2.

**Table S1.** Excel file containing the qRT‐PCR primers for reference and target genes used to characterize MCF7 iZEB cells.

**Table S2.** Excel file containing the Pearson correlation coefficients for each replicate per cell line after log_2_ transformation. Green boundaries are the values between PANC‐1 replicates, blue for CAPAN‐1 and orange for MIA PaCa‐2. Red boundaries indicate the unexpected high correlation between PANC‐1 replicates and MIA PaCa‐2 replicate 1.

**Table S3.** Excel file containing the results of the ANOVA test and the pairwise comparison (two‐sample *t*‐test) between the pancreatic cancer cell lines.

**Table S4.** Excel file containing the results of the overrepresentation analysis (Fig. S7).

**Table S5.** Excel file containing the counts of the control wells and calculations of the recovery rates (Fig. 5).

## Data Availability

The mass spectrometry proteomics data have been deposited to the ProteomeXchange Consortium in the PRIDE [48] partner repository with the dataset identifier PXD057259.
