# Supplementary material for: EMT‐associated bias in the Parsortix® system observed with pancreatic cancer cell lines
Source: Mol Oncol. 2025 Jun 18;19(11):3360–75. doi: 10.1002/1878-0261.70066 (PMC12591324; doi:10.1002/1878-0261.70066)
Supplement: Supplementary file 1 — Fig. S1. Quality control of the label‐free quantification mass spectrometry data of all the pancreatic cancer cell lines CAPAN‐1, PANC‐1 and MIA PaCa‐2 with the first replicate of MIA PaCa‐2 omitted. Fig. S2. CDH1 expression of extracellular CDH1 on the CAPAN‐1, PANC‐1 and MIA PaCa‐2 pancreatic cancer cell lines by flow cytometry. Fig. S3. Expression of pan‐cytokeratins (pan‐CKs) in the CAPAN‐1, PANC‐1 and MIA PaCa‐2 pancreatic cancer cell lines. Fig. S4. Analysis of expression of mesenchymal/epithelial markers fibronectin‐1 (FN1) and EPCAM in the CAPAN‐1, PANC‐1 and MIA PaCa‐2 pancreatic cancer cell lines. Fig. S5. Analysis of expression of epithelial/mesenchymal markers claudin‐4 (CLDN4) and N‐cadherin (CDH2) in the CAPAN‐1, PANC‐1 and MIA PaCa‐2 pancreatic cancer cell lines. Fig. S6. Analysis of expression of mesenchymal marker CD44 in the CAPAN‐1, PANC‐1 and MIA PaCa‐2 pancreatic cancer cell lines. Fig. S7. Differential expression analysis between the pancreatic cell lines: CAPAN‐1, PANC‐1 and MIA PaCa‐2. [file MOL2-19-3360-s004.docx]

**Supplementary Figures**

**
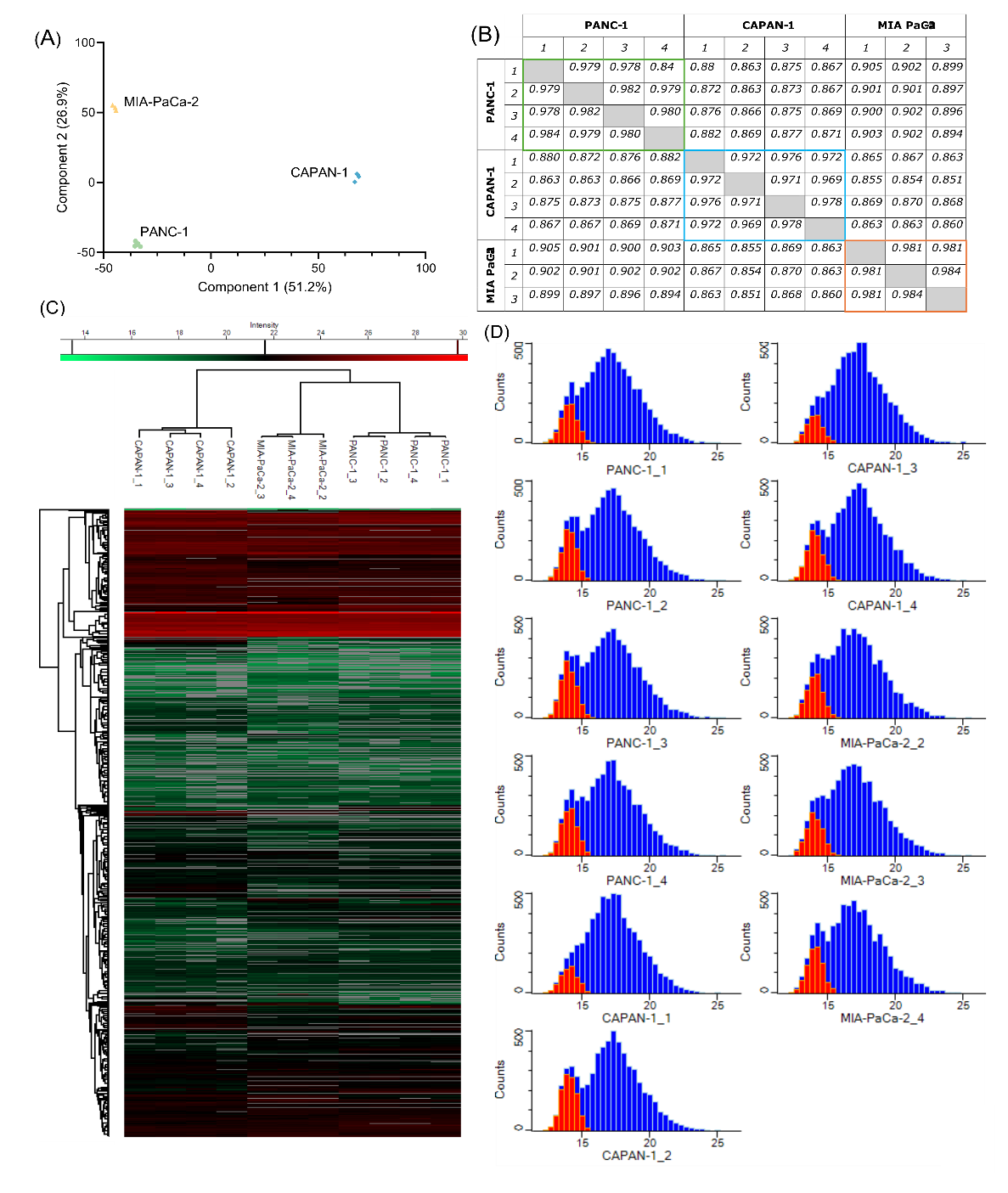
**

**Supplementary Figure 1s:** Quality control of the label-free quantification mass spectrometry data of all the pancreatic cancer cell lines CAPAN-1, PANC-1 and MIA PaCa-2 with the first replicate of MIA PaCa-2 omitted. (A) Principal component analysis of each replicate per cell line after log2transformation. (B) Pearson correlation coefficients for each replicate per cell line after log2 transformation. (C) Heatmap of all samples before imputation. Protein groups with high intensities are red, low intensities are blue and missing values are grey. (D) Histograms showing the distribution of intensity values after imputation (substituted values shown in red).


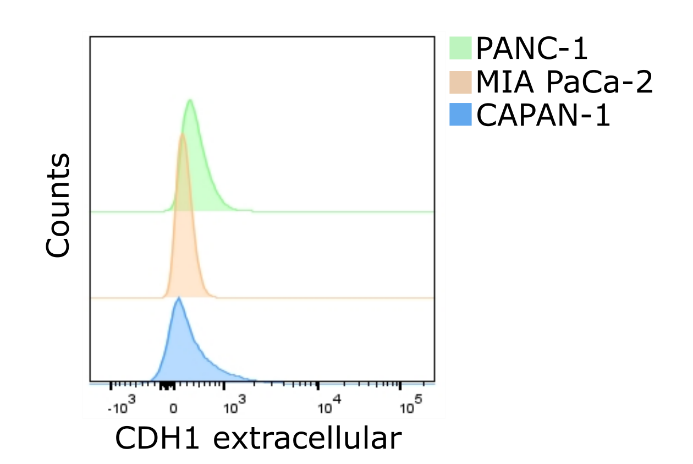


**Supplementary Figure 2s:** CDH1 expression of extracellular CDH1 on the CAPAN-1, PANC-1 and MIA PaCa-2 pancreatic cancer cell lines by flow cytometry.


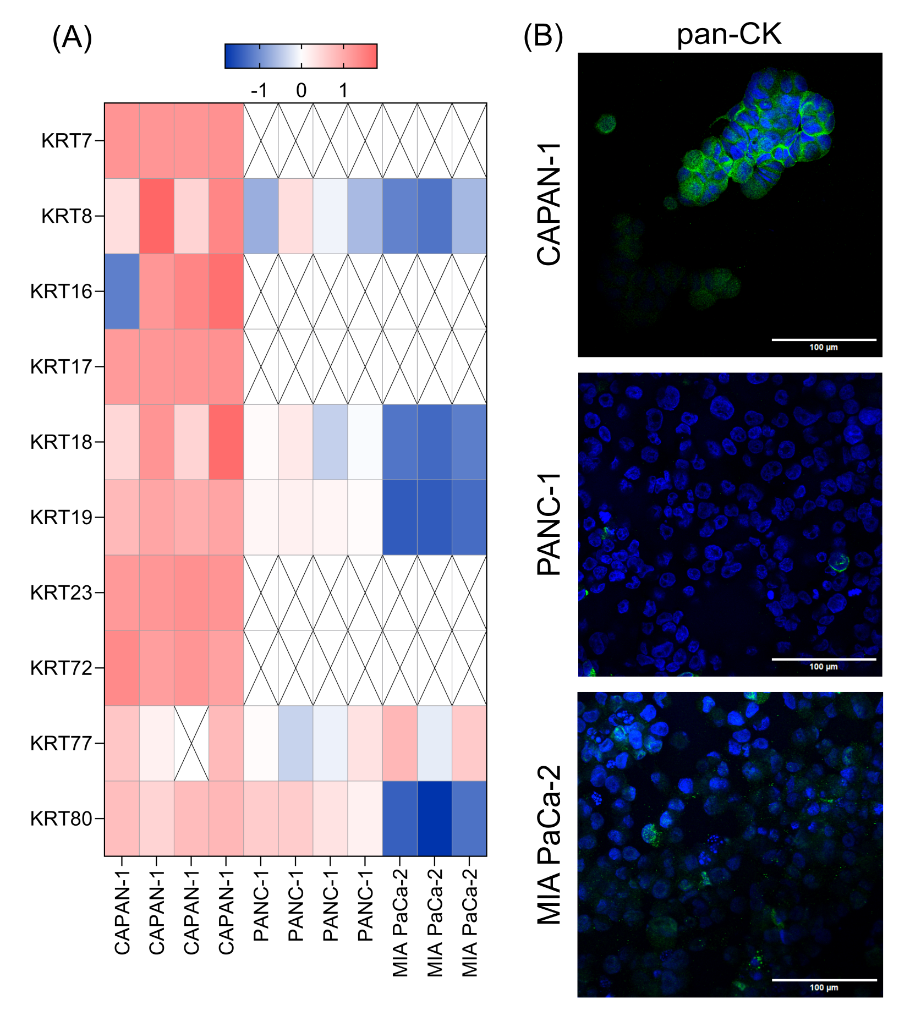


**Supplementary Figure 3s:** Expression of pan-cytokeratins (pan-CKs) in the CAPAN-1, PANC-1 and MIA PaCa-2 pancreatic cancer cell lines by (A) mass spectrometry (z-scores are plotted in a heatmap). Red cells indicate a high Z-score, blue cells a low Z-score, empty cells represent samples where the protein was not detected. (B) Expression of pan-CK was evaluated by immunofluorescent staining. DAPI (blue) was used as nuclear stain. Scale bar: 100 µm.

*
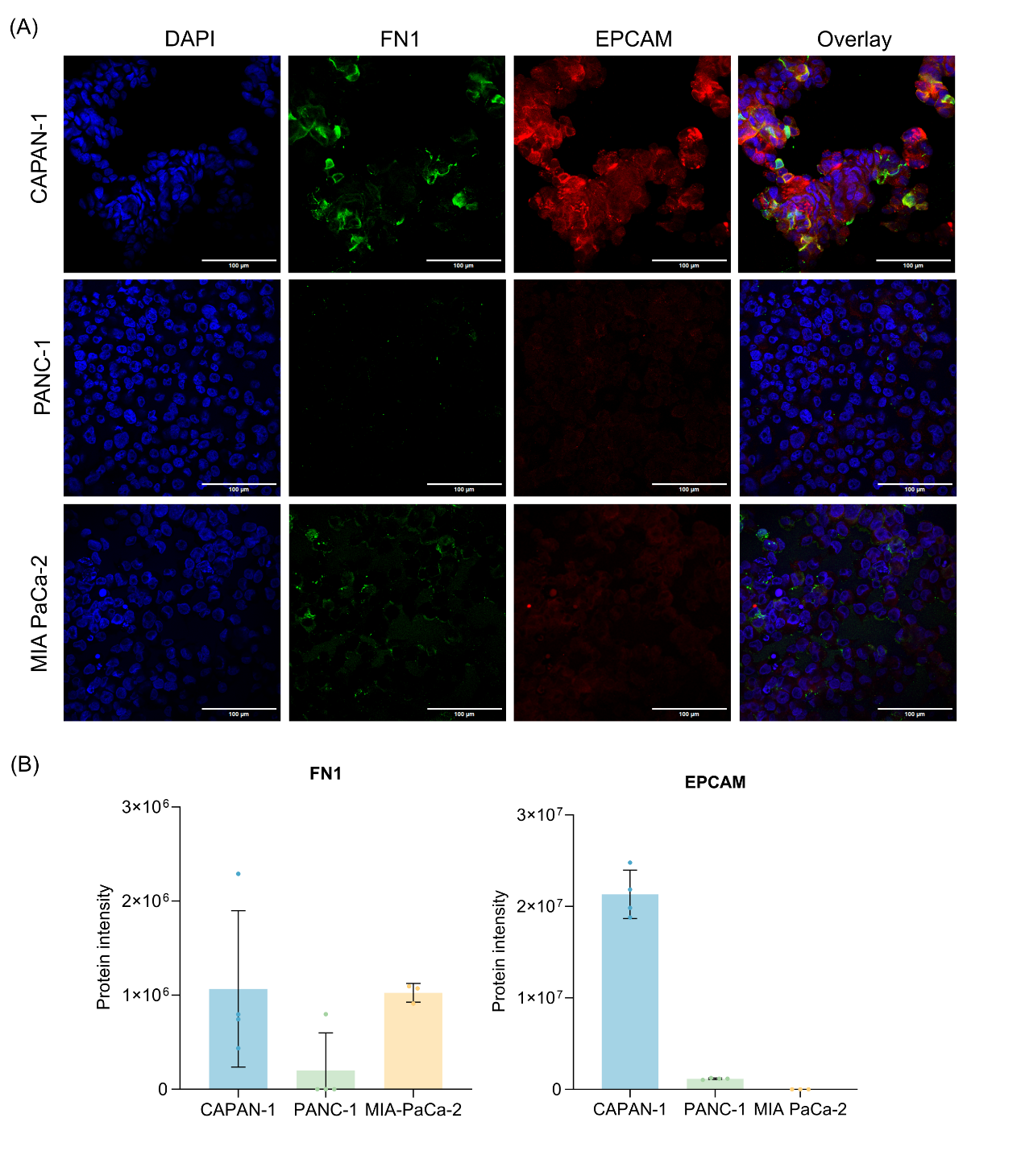
*

**Supplementary Figure 4s:** Analysis of expression of mesenchymal/epithelial markers fibronectin-1 (FN1) and EPCAM in the CAPAN-1, PANC-1 and MIA PaCa-2 pancreatic cancer cell lines (A) Expression of FN1 and EPCAM was evaluated by immunofluorescent staining. DAPI (blue) was used as nuclear stain. Scale bar: 100 µm. (B) Protein intensities of FN1 and EPCAM based on the mass spectrometry data from the pancreatic cancer cell lines: CAPAN-1, PANC-1 and MIA PaCa-2, without log2 transformation and without imputed values instead missing values were replaced with a zero value.


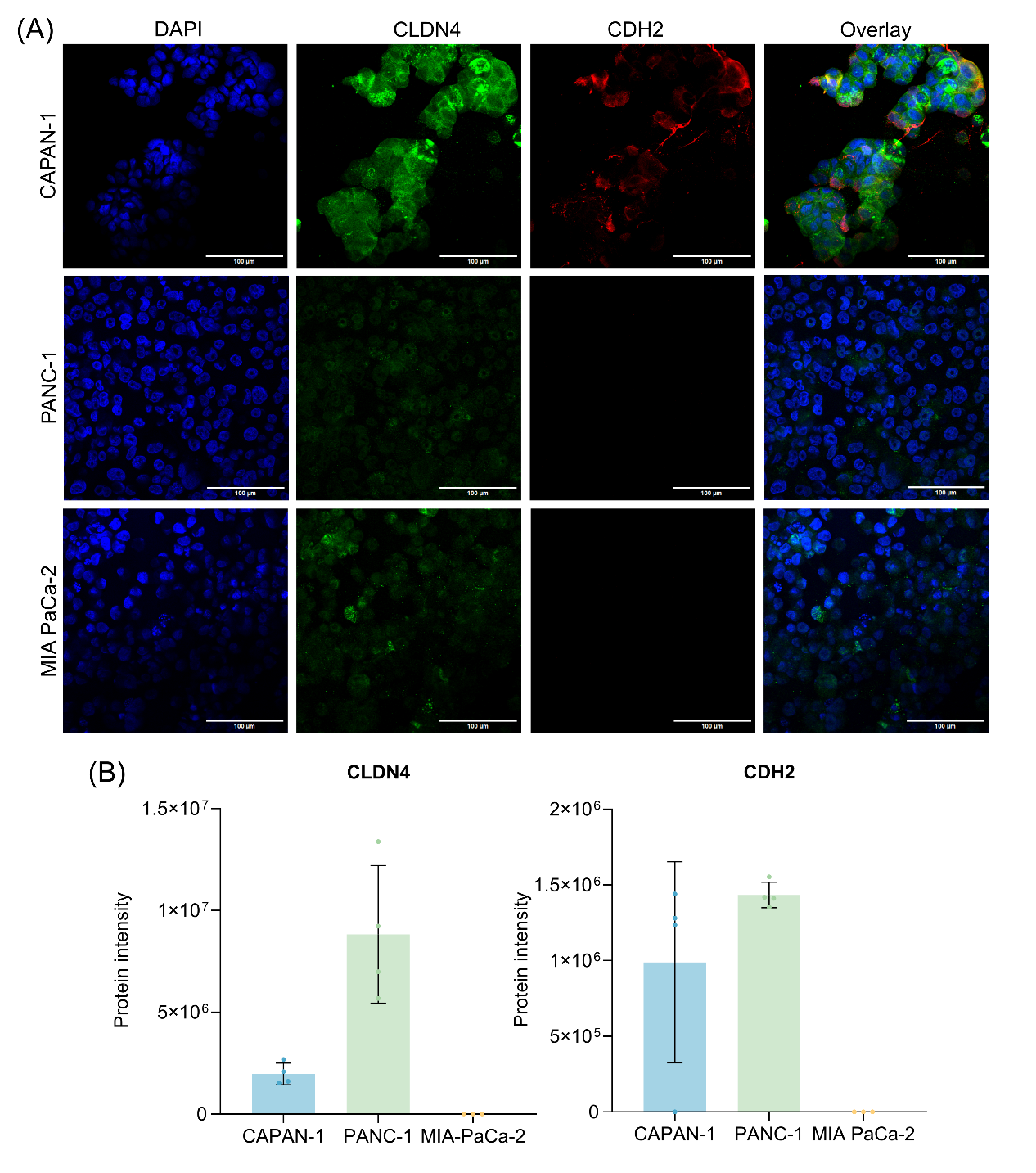


**Supplementary Figure 5s:** Analysis of expression of epithelial/mesenchymal markers claudin-4 (CLDN4) and N-cadherin (CDH2) in the CAPAN-1, PANC-1 and MIA PaCa-2 pancreatic cancer cell lines (A) Expression of CLDN4 and CDH2 was evaluated by immunofluorescent staining. DAPI (blue) was used as nuclear stain. Scale bar: 100 µm. (B) Protein intensities of CLDN4 and CDH2 based on the mass spectrometry data from the pancreatic cancer cell lines: CAPAN-1, PANC-1 and MIA PaCa-2, without log2 transformation and without imputed values instead missing values were replaced with a zero value.

**
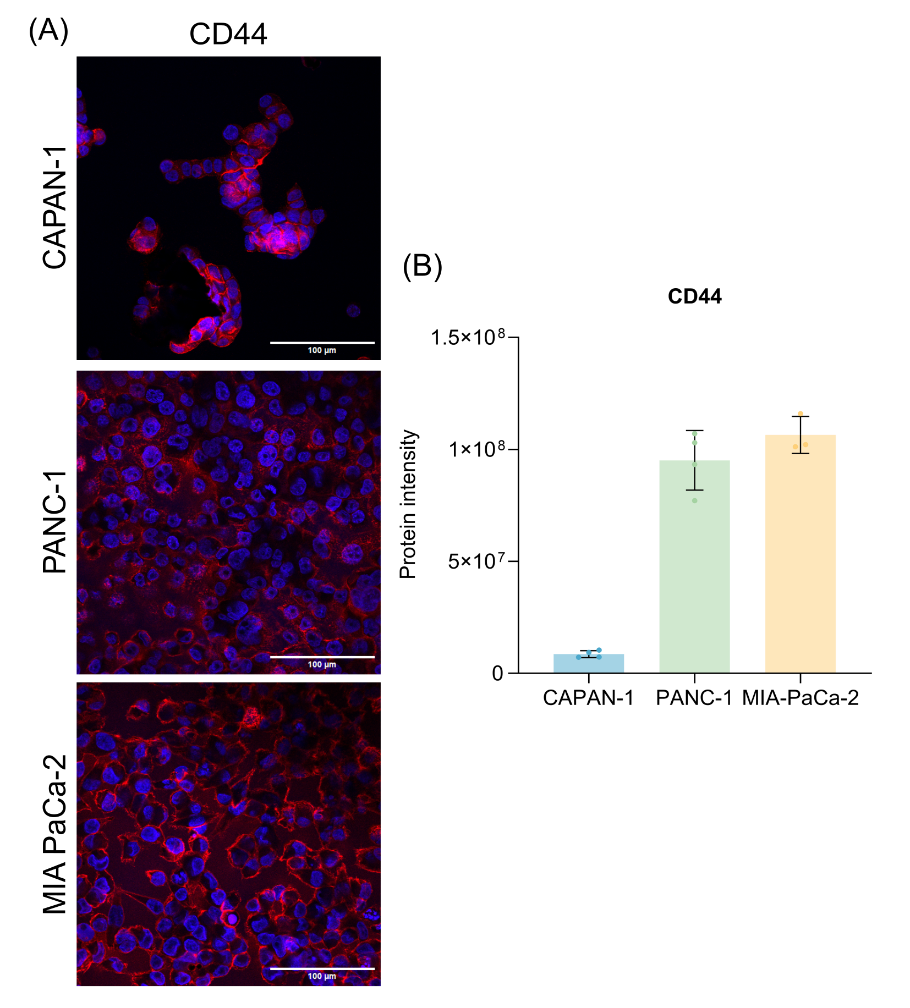
**

**Supplementary Figure 6s:** Analysis of expression of mesenchymal marker CD44 in the CAPAN-1, PANC-1 and MIA PaCa-2 pancreatic cancer cell lines (A) Expression of CD44 was evaluated by immunofluorescent staining. DAPI (blue) was used as nuclear stain. Scale bar: 100 µm. (B) Protein intensities of CD44 based on the mass spectrometry data from the pancreatic cancer cell lines: CAPAN-1, PANC-1 and MIA PaCa-2, without log2 transformation and without imputed values instead missing values were replaced with a zero value.

**
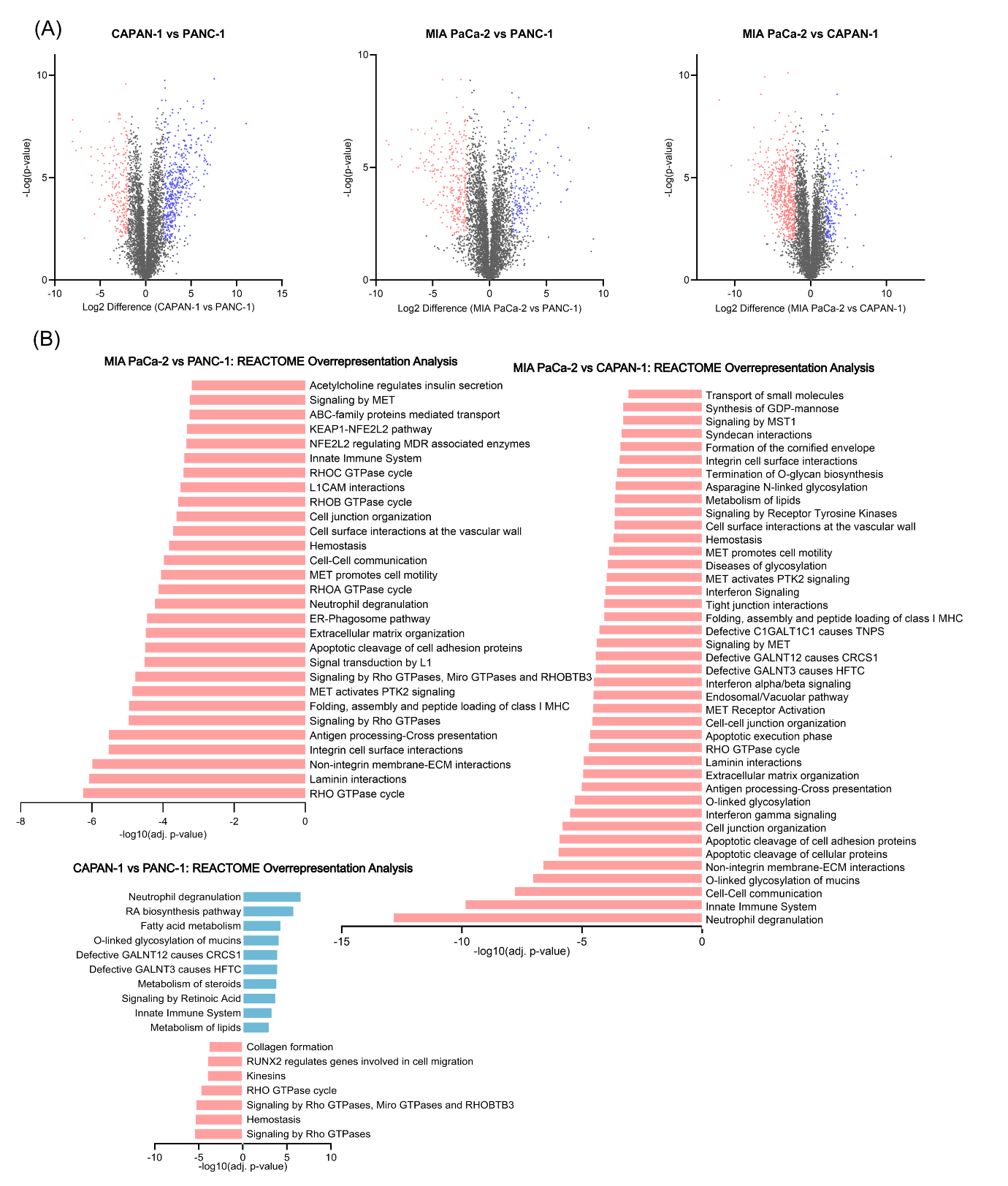
**

**Supplementary Figure 7s:** Differential expression analysis between the pancreatic cell lines: CAPAN-1, PANC-1 and MIA PaCa-2. (A) Volcano plots of differentially expressed proteins between the three pancreatic cancer cell lines. Red dots represent the significantly downregulated proteins (p < 0.01 and log2(FC) < -2), blue dots represent the significantly upregulated proteins (p < 0.01 and log2(FC) > 2), the grey dots are proteins that were unsignificant or have a low fold change (p > 0.01 or log2(FC) < 2 or > -2). (B) Overrepresentation analysis of REACTOME pathways of differentially significant (p < 0.01) proteins of CAPAN-1 vs PANC-1, MIA PaCa-2 vs CAPAN-1 and MIA PaCa-2 vs PANC-1. The pathways indicated in red are overrepresented in the significantly downregulated proteins (log2(FC) < -2), the pathways in blue are overrepresented in the significantly upregulated proteins (log2(FC) > 2).
